# Evaluation of a minimal sedation protocol using ICU sedative consumption as a monitoring tool: a quality improvement multicenter project

**DOI:** 10.1186/s13054-014-0580-3

**Published:** 2014-10-24

**Authors:** Otavio T Ranzani, Evelyn Senna Simpson, Talita Barbosa Augusto, Sylas Bezerra Cappi, Danilo Teixeira Noritomi, on behalf of the AMIL Critical Care Group

**Affiliations:** Critical Care Department, Hospital Paulistano, Rua Martiniano de Carvalho, 741, São Paulo, 01321-001 Brazil; Respiratory Intensive Care Unit, Pulmonary Division, Heart Institute, Hospital das Clínicas, University of São Paulo, Rua Enéas Carvalho de Aguiar, 255, sala 6040, 6° andar, São Paulo, 05403-000 Brazil

## Abstract

**Introduction:**

Oversedation frequently occurs in ICUs. We aimed to evaluate a minimal sedation policy, using sedative consumption as a monitoring tool, in a network of ICUs targeting decrement of oversedation and mechanical ventilation (MV) duration.

**Methods:**

A prospective quality improvement project was conducted in ten ICUs within a network of nonteaching hospitals in Brazil during a 2-year period (2010 to 2012). In the first 12 months (the preintervention period), we conducted an audit to identify sedation practice and barriers to current guideline-based practice regarding sedation. In the postintervention period, we implemented a multifaceted program, including multidisciplinary daily rounds, and monthly audits focusing on sedative consumption, feedback and benchmarking purposes. To analyze the effect of the campaign, we fit an interrupted time series (ITS). To account for variability among the network ICUs, we fit a hierarchical model.

**Results:**

During the study period, 21% of patients received MV (4,851/22,963). In the postintervention period, the length of MV was lower (3.91 ± 6.2 days versus 3.15 ± 4.6 days; mean difference, −0.76 (95% CI, −1.10; −0.43), *P* <0.001) and 28 ventilator-free days were higher (16.07 ± 12.2 days versus 18.33 ± 11.6 days; mean difference, 2.30 (95% CI, 1.57; 3.00), *P* <0.001) than in the preintervention period. Midazolam consumption (in milligrams per day of MV) decreased from 329 ± 70 mg/day to 163 ± 115 mg/day (mean difference, −167 (95% CI, −246; −87), *P* <0.001). In contrast, consumption of propofol (*P* = 0.007), dexmedetomidine (*P* = 0.017) and haloperidol (*P* = 0.002) increased in the postintervention period, without changes in the consumption of fentanyl. Through ITS, age (*P* = 0.574) and Simplified Acute Physiology Score III (*P* = 0.176) remained stable. The length of MV showed a secular effect (secular trend β_1_ = −0.055, *P* = 0.012) and a strong decrease immediately after the intervention (intervention β_2_ = −0.976, *P* <0.001). The impact was maintained over the course of one year, despite the waning trend for the intervention’s effect (postintervention trend β_3_ = 0.039, *P* = 0.095).

**Conclusions:**

By using a light sedation policy in a group of nonteaching hospitals, we reproduced the benefits that have previously been demonstrated in controlled settings. Furthermore, systematic monitoring of sedative consumption should be a feasible instrument for supporting the implementation of a protocol on a large scale.

**Electronic supplementary material:**

The online version of this article (doi:10.1186/s13054-014-0580-3) contains supplementary material, which is available to authorized users.

## Introduction

The pharmacologic control of analgesia and sedation is an almost ubiquitous routine in everyday practice in intensive care units (ICUs) worldwide, especially in the management of symptoms of mechanically ventilated patients [1,2]. Sedation is used to relieve discomfort, control agitation and anxiety and help in the management of critical states. However, oversedating or maintaining redundant pharmacologic medications occurs frequently and is associated with short- and long-term adverse events [2-8]. Importantly, the degree of sedation and the use of certain types of medications, such as benzodiazepines, are associated with poor clinical outcomes, including augmented duration of mechanical ventilation (MV), increased length of stay in the hospital and even mortality [2-4,9-12].

To overcome these problems, several authors have employed different methods to deliver a more suitable sedation regimen, such as goal-directed sedation [13]; daily interruption [5]; protocolized, nurse-driven sedation [14]; and minimized use of continuous infusions [6,15]. These authors reported an improvement in the offered sedation and analgesia practice and a decrease in the incidence of adverse events compared to control groups. Nevertheless, controversy still exists because several other authors have reported neutral results [16-20]. In fact, even assuming the likelihood of benefits that might be associated with the reduction of sedatives, the current literature involving nonexperimental settings suggests that there are several barriers to implementing strategies aimed at improving the delivered sedation [21-25]. Additionally, the use of resources associated with this type of initiative cannot be neglected.

We developed a simple minimal sedation protocol and a reliable monitoring tool based on the consumption of sedatives in the entire ICU. During the course of 1 year, we employed this strategy in a group of ten ICUs in private nonteaching hospitals. The objective of our study was to verify whether this strategy results in benefits similar to those previously reported in more controlled scenarios.

## Material and methods

### The scenario

The Amil Critical Care Group consists of 12 ICUs (total of 200 ICU beds) in a group of 11 hospitals associated with a health maintenance organization (HMO) in Sao Paulo, Brazil. Two of the authors (DTN and ESS)—a doctor and a nurse who are responsible for the group’s policy making, implementation and monitoring of common routines—coordinate this group. The light sedation protocol was implemented earlier in two ICUs in the Amil Critical Care Group during a pilot phase coordinated by three of the authors (DTN, ESS and SBC). All of the data obtained from these two ICUs were excluded from the present analysis, resulting in ten ICUs ultimately being analyzed. Their main characteristics are provided in Table 1.

**Table 1:** **General characteristics of ten intensive care units analyzed in the quality improvement project** ^**a**^

**Unit ID**	**Type of ICU**	**ICU beds**	**ICU structure**	**Hospital beds**	**Major type of admission diagnosis**	**Use of invasive mechanical ventilation among admitted patients** ^**b**^
1	Mixed	19	Closed	119	General	15.1%
2	Cardiologic	19	Closed	119	Cardiologic	35.7%
3	Mixed	10	Closed	131	Respiratory	20.2%
4	Mixed	10	Closed	82	Respiratory	18.3%
5	Mixed	11	Closed	150	Respiratory	19.7%
6	Mixed/cardiologic	36	Closed	206	Respiratory	26.6%
7	Mixed	10	Closed	233	Respiratory	22.1%
8	Mixed	12	Closed	77	Respiratory	4.9%
9	Mixed	6	Closed	227	Orthopedic	20.0%
10	Neurologic	19	Closed	227	Neurologic	17.4%

^a^ICU, Intensive care unit. ^b^Mean rate during the 2-year period of the study.

The Research and Ethics Committee of Hospital Pró-Cardíaco, which is the reference ethics committee designated by the National Research Ethics Committee, approved the retrospective analysis and publication of the data on behalf of the entire network under the number 387.388 and waived the need for informed consent.

### Protocol development

Our intervention was conducted over the course of a 12-month period from October 2011 to October 2012 through multidisciplinary meetings. This protocol is part of our quality improvement (QI) program [26], which involves all of the care of acutely ill patients (for example, sepsis management [27], hospital-acquired infections).

We established a baseline period (October 2010 to October 2011) during which a monthly audit was conducted, and we retrieved information concerning installed sedation protocols, knowledge of the minimal sedation concept and barriers to implementation. In this period, we also reviewed the amount of sedative consumption.

**Figure 1:**
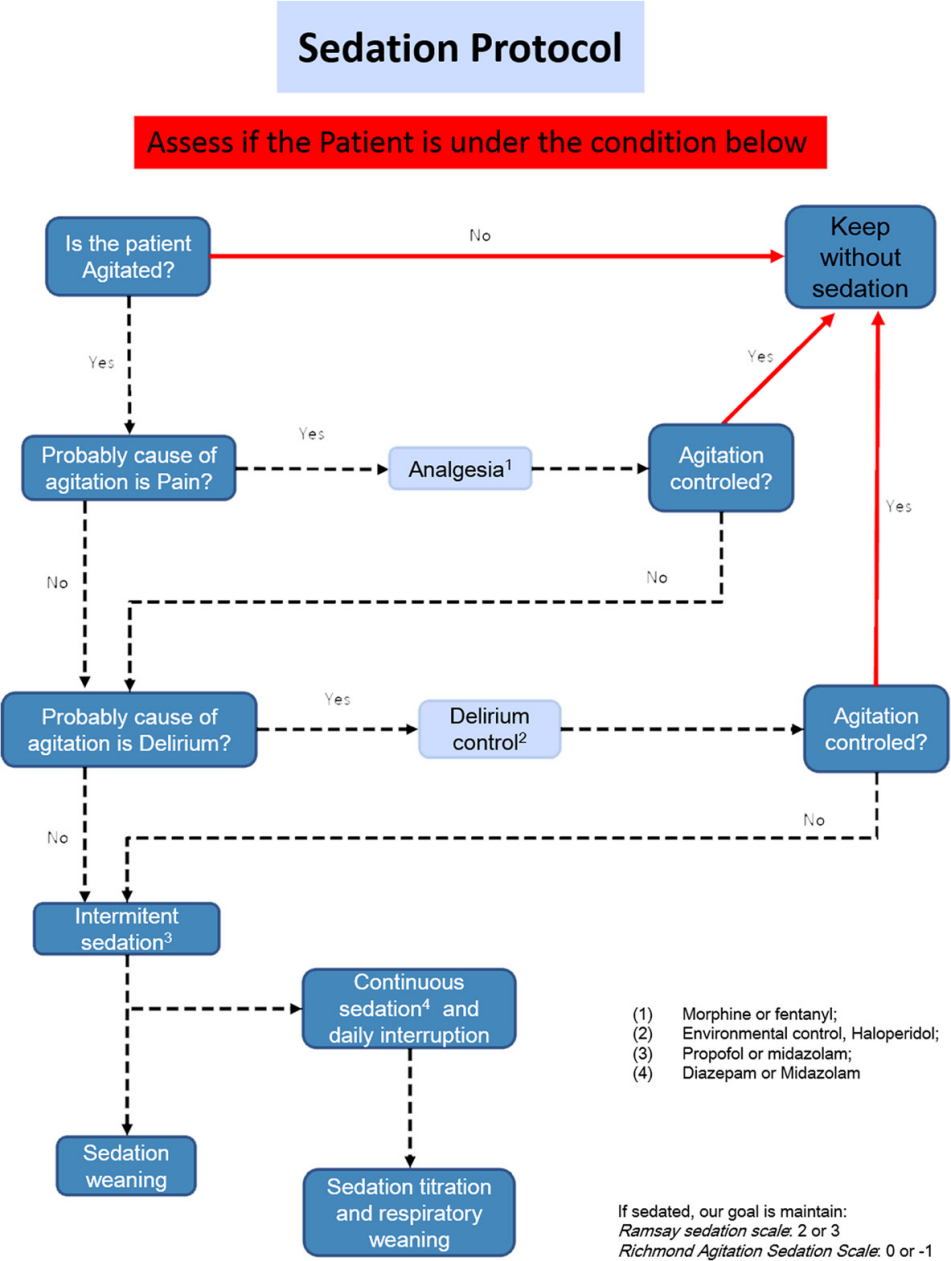
**The minimal sedation protocol algorithm.**

Rather than detailing the definitive pathways for sedation, we chose to define comprehensive guidelines, which are summarized in Figure 1. These guidelines primarily state that a patient does not need to be continuously sedated unless patient discomfort persists after assessing and treating pain, delirium and after a trial of low-dose, intermittent sedation has been performed. The protocol includes a formal suggestion to practice daily sedation interruption, which was driven by the assistant nurse. If drugs were continuously infused, nurses assessed the patient sedation level hourly and whether it was outside the goal (Richmond Agitation Sedation Scale score of 0 or −1/Ramsay sedation scale score of 2 or 3), the dosage infusion was discussed with the attending physician.

The light sedation guidelines were not applied for patients during the acute phase of selected conditions (for example, traumatic brain injury), for patients receiving neuromuscular blocking agents or when the guidelines were not indicated by the attending physician.

### Protocol implementation and monitoring

The overall design of the intervention was based on the model presented by previous authors (“knowledge-to-action”) [28-30], which describes the necessary steps for implementation of knowledge and discusses the nature of its barriers. The initial barrier assessment was performed by inputs from the multidisciplinary team in several participating ICUs. The successful experience of two units from our group was also taken into account to identify barriers and plan actions.

The components of the intervention are described in Table 2. The change strategies were common across participating sites (that is, audit and feedback, educational outreach, performance coaching, opinion leaders, networking meeting), even though they could differ in details; for example, the opinion leader could be the head of the ICU or one of the regular staff doctors. In brief, protocol implementation was started after a formal lecture session with the leaders of all the ICUs (doctors, nurses, respiratory therapists and clinical pharmacists). In the lecture session, the rationale of minimal sedation was described, the guidelines were presented and the results of our pilot experience were discussed. In this lecture and following meetings, the protocol (Figure 1) was shown and reinforced.

**Table 2:** **Framework for a minimal sedation protocol implementation** ^**a**^

**Nature of barrier**	**Barrier**	**Intervention Categorization**	**Description**	**Comments**
Knowledge	Lack of familiarity	Interactive educational session	An evidence-based review of the benefits of minimal sedation and the guidelines were shown.	In this section, the first concerns and questions about the minimal sedation policy could be discussed.
		Educational outreach visit	Random bedside rounds accompanied by two of the authors responsible for the coordination of the group of ICUs	Mechanically ventilated patients were identified and the possibility of minimal sedation institution was discussed individually for each patient.
	Lack of awareness; commonly, doctors state they already use ideal sedation	Initial benchmarking	The range of outcome (length of mechanical ventilation and sedative consumption) was demonstrated to all the ICU leaders.	There was wide variability among ICUs, suggesting there was an opportunity for improvement.
	Lack of self-efficacy	Use of early adopters’ example	The experience of one unit and its methods used to overcome barriers were shown to all other ICU leaders.	One of the least resourced ICUs was the first to obtain positive results.
	Lack of self-efficacy	Performance coaching	Monthly/weekly feedback concerning sedative consumption and length of mechanical ventilation	In selected cases, weekly feedback was given, with identification of specific days of larger sedative consumption (mostly on weekends).
Attitude	Lack of agreement	External validation	Knowledgeable doctor was invited to give the initial presentation.	The credibility of the proposed policy was endorsed by an academic leader.
Behavior	Conflict among the multidisciplinary team	Definition of common goals	Multidisciplinary involvement in meetings and bedside rounds	Nurses, respiratory therapists, clinical pharmacists and physicians were encouraged to get involved in the discussion of sedation goals at rounds.

^a^ICU, Intensive care unit.

The leaders in each unit were responsible for implementing the routine. To empower the campaign of light sedation, we used the daily multidisciplinary rounds that had already taken place in all of the units, and we encouraged use of this structure to disseminate the protocol and discuss its implementation [26]. In the daily multidisciplinary rounds, we update a portion of the applied checklist to be dedicated to sedation and MV topics. (Is it possible to wean the patient from MV? Is the patient under continuous sedation? Is the target sedation achieved? If deeply sedated, was sedation interrupted this morning? Is there adequate control of pain and agitation?) There were monthly meetings with our team and the leaders of each unit, at which time the leaders were invited to present their results and share their experiences in overcoming barriers.

Considering that certain classes of medications, such as the benzodiazapines (for example, midazolam), are used primarily for sedating MV patients, we decided to use the entire ICU consumption of such medications as an indicator of protocol compliance, assuming that the lesser the amount of medication that is consumed, the higher the adherence to our minimal sedation guidelines. Furthermore, we used the total consumption in milligrams, corrected by both the number of patients who were admitted during the period and the mean duration of MV. This indicator has the following obvious advantages over the traditional processes (that is, direct observation of daily arousal or down-titration of sedatives): It is an observer-independent parameter, easy to obtain, not susceptible to the Hawthorne effect and potentially associated with the desired outcome. Although some case mix–related variation could occur, this effect would be minimized over time.

The results of the consumption of the primary sedatives and analgesics available were shared in every monthly meeting for each ICU in the group. As part of the QI project in the HMO, benchmarking between ICUs was used. Additionally, the duration of MV was presented and discussed for each ICU.

### Data collection method

The data were retrieved from a clinical database that has been in place since 2009 in three ICUs and since 2010 in all of the units. This database was audited by a specialized private company (EPIMED solutions, Rio de Janeiro, Brazil) and maintained by a dedicated staff from the Amil Critical Care Group HMO. Each hospital had a case manager (nurse), who was committed exclusively to inputting data daily into a case report formulary (CRF) using the Epimed Monitor system software (EPIMED solutions). Each record was tagged using a unique identifier for each patient without releasing personal data to maintain patient anonymity.

The database maintains a myriad of controls to guarantee the quality of the recorded data. Definitions were clearly stated in the CRF and were also available in a sheet of guidelines. To ensure reasonable inspection and to avoid misspecifications and typographical errors, several variables were assigned a real range. Furthermore, general information was entered with no manual transcription using an electronic system employed in our ICUs.

Random continuous checks and audits of the ongoing data collection were meticulously screened by two investigators (DTN and ESS) for missing information, implausible and outlying values, logical errors and insufficient details. A third investigator (OTR) performed similar, frequent, retrospective offline audits of the exported data and provided feedback to the group.

The absolute consumption of prespecified sedoanalgesic medications (midazolam, propofol, dexmedetomidine, fentanyl and haloperidol) per unit per month was retrieved from the local pharmacy in each unit, which is the department responsible for all of the medications used in the unit. Each local pharmacy is under the management of a central pharmacy in each hospital.

### Statistical analysis

The data were tested for normal distribution using histograms and the Kolmogorov-Smirnoff test or Shapiro-Wilk test as appropriate. The categorical and continuous data are presented as percentages and mean ± SD values (or medians and interquartile ranges (IQR), respectively. The categorical variables were compared using a χ^2^ test or Fisher’s exact test as appropriate. The quantitative continuous variables were compared using an unpaired *t*-test and the Mann–Whitney *U* test for normally and non-normally distributed variables, respectively.

To analyze the effect of the campaign, we planned a quasi-experimental design using interrupted time series (ITS) to control for secular trends [31-33]. The ITS design is the strongest quasi-experimental design for evaluating the effects of time-delimited interventions. The data were collected at multiple instances over time, before and after an intervention, to detect whether the intervention had a significantly greater effect than the underlying secular trend. An advantage of the ITS design is that it can adjust for time trends, estimate changes in time trends and account for autocorrelation. Furthermore, it allows the investigation of potential biases together with the secular trend, such as the duration of the intervention (that is, that the intervention might have an effect for the first 2 months only, rather than a sustained effect) and seasonal or cyclical effects.

To model the effect of the campaign, we run autoregressive integrated moving average (ARIMA) models. Two parameters define each segment (before and after the intervention) of a time series: level and trend. The level is the value of the series at the beginning of a given time interval. The trend is the rate of change of a measure (slope) during a segment. To examine the results, we might analyze whether there are changes in level and trend that follow an intervention. In general, a change in level constitutes an immediate intervention effect, and a change in trend represents a gradual variation in the outcome.

Multiple time series require different approaches, which consist of assessing not only the absolute value but also the shape of each series. To enhance our analysis while correctly addressing our multicenter data, we performed two approaches for our main outcomes (length of MV and midazolam consumption). First, patient data were aggregated by month. In one approach, we retrieved an average value per month representing the network, which was an arithmetic mean value per unit per month. In another approach, we fit a hierarchical time series [34] by using a bottom-up method. This approach involves first providing independent time series at the bottom level of the hierarchy (each ICU, level 1) and then aggregating the independent time series upward to produce a revised time series for the whole hierarchy (network, level 0). This method accounts for noise and variability between time series and provides additional information in comparison to a crude average.

To deal with variation in case mixes over time at two levels (patient and unit), we conducted a sensitivity analysis for our main outcome fitting a generalized linear mixed model, achieving the better fit for correlated responses, in particular for the analysis of our longitudinal and clustered data. The length of MV for each patient was the dependent variable. For the first level (patient level), we adjusted for (Simplified Acute Physiology Score III (SAPS III), Charlson index score and vasoactive drugs. For the second level (unit level), we fit random intercepts for each one of ten units and nested into the variable units random components for type of admission and reasons for admitting: sepsis syndrome, cardiac surgery and respiratory conditions. The model was built with a Poisson distribution with a log link function, and the covariance structure for the random effects was first-order autoregressive [35,36].

We evaluated the association between midazolam consumption and length of MV fitting a linear mixed model. The monthly consumption of midazolam adjusted per number of mechanically ventilated patients per each ICU was the dependent variable. The length of MV per month per each ICU was the outcome evaluated. In the model, both variables were transformed with the use of their natural logarithms to reduce the influence of extreme outliers. To adjust for severity, we entered SAPS III score as a covariate. We used splines to allow nonlinear associations between midazolam and length of MV [9,37].

*P* <0.05 was considered to be statistically significant for all of the analyses. The R free source statistical package version 2.15.2 ([38]; The R Project for Statistical Computing, Vienna Austria), CRAN ([39])-specific libraries and the SPSS 19.0 package for Windows software (IBM SPSS, Chicago, IL, USA) were used in all of the analyses.

Further information on the statistical analysis plan is available in Additional file 1.

## Results

We analyzed all of the adult patients admitted to ten ICUs between October 2010 and October 2012. There were 22,963 admissions during this period, which included 11,256 between October 2010 and September 2011, defined as the preintervention period, and 11,709 admissions between October 2011 and October 2012, defined as the postintervention period.

### Comparison between the pre- and postintervention periods

There were no significant differences in relation to sex or age between the two groups. There was a slight difference in SAPS III scores between periods, with higher severity in the preintervention period (42.5 ± 15 vs. 42.1 ± 15, *P* = 0.027). The types of admission were similar between the groups. There were predominantly medical admissions (74%), and cardiovascular was the most prevalent diagnostic category for both periods. Sepsis was more prevalent in the postintervention period (12.3% vs. 14.1%, *P* <0.001), whereas the respiratory category was more prevalent in the preintervention period (6.9% vs. 5.5%, *P* <0.001). Need MV during the ICU stay were slightly more frequent in the preintervention period (21.7% vs. 20.5%, *P* = 0.027), but the percentages of patients who were receiving MV at the time of admission were not significantly different (14.9% vs. 14.3%, *P* = 0.190) (Table 3).

**Table 3:** **Characteristics of overall cohort** ^**a**^

	**Preintervention (** ***n*** **= 11,256)**	**Postintervention (** ***n*** **= 11,709)**	***P*** **-value**
**Characteristics**	**October 2010 to September 2011**	**October 2011 to October 2012**	
Age, yr, mean ± SD	59.62 ± 19	59.53 ± 19	0.714
Male, *n* (%)	5,705 (50.7)	5,924 (50.6)	0.891
BMI, kg/m^2^, mean ± SD	26.9 ± 6	27.1 ± 6	0.055
SAPS III score, mean ± SD	42.49 ± 15	42.06 ± 15	0.027
Charlson index score, median (IQR)	1 (0; 2)	1 (0; 2)	0.106
Admission type, *n* (%)			0.021
Medical	8,399 (74.6)	8,549 (73.0)	
Elective surgical	2,547 (22.6)	2,285 (24.1)	
Emergency surgical	310 (2.8)	333 (2.8)	
Diagnosis category, *n* (%)			
Cardiovascular	3,390 (30.1)	3,480 (29.7)	0.512
Sepsis	1,386 (12.3)	1,653 (14.1)	<0.001
Neurological	1,376 (12.2)	1,336 (11.4)	0.056
Respiratory^b^	778 (6.9)	645 (5.5)	<0.001
Orthopedic surgery	733 (6.5)	814 (7.0)	0.184
Neurosurgery	218 (1.9)	267 (2.3)	0.070
Thoracic surgery	89 (0.8)	99 (0.8)	0.645
Cardiac surgery	625 (5.6)	706 (6.0)	0.122
Resources during ICU, *n* (%)			
RRT^c^	410 (3.6)	414 (3.5)	0.665
Vasopressors^c^	1,838 (16.3)	2,055 (17.6)	0.013
Noninvasive ventilation^c^	1,735 (15.4)	1,314 (11.2)	<0.001
Mechanical ventilation, *n* (%)			
During ICU stay	2,446 (21.7)	2,405 (20.5)	0.027
At ICU admission	1,677 (14.9)	1,673 (14.3)	0.190
ICU LOS, days			
Mean ± SD	4.20 ± 6.5	3.87 ± 5.3	<0.001
Median (IQR)	2 (1; 4)	2 (1; 4)	0.095
Total hospital LOS, days			
Mean ± SD	13.29 ± 22.6	12.94 ± 20.1	0.215
Median (IQR)	7 (4; 14)	7 (4; 14)	0.110
Mortality, % (95% CI)			
ICU mortality	9.5% (9.0; 10.0)	7.3% (6.8; 7.7)	<0.001
Hospital mortality	14.1% (13.5; 14.8)	11.9% (11.3; 12.5)	<0.001

^a^BMI, Body mass index; ICU, Intensive care unit; LOS, Length of stay; MV, Mechanical ventilation; RRT, Renal replacement therapy; SAPS III, Simplified Acute Physiology Score III. ^b^Respiratory diagnosis except pneumonia. ^c^Data were missing for two patients.

In analyzing 4,851 patients (21%) who were under MV during their ICU stay (Table 4), we observed that the group characteristics were similar with regard to age and sex. However, we detected a minor difference in SAPS III scores; the less acutely ill patients were in the postintervention period (52.7 ± 19 vs. 51.4 ± 20, *P* = 0.031). Despite their lower mean SAPS III scores, the postintervention group had more patients who used vasopressors during their ICU stay (16.3% vs. 17.6%, *P* <0.001). Patients who underwent MV in the postintervention period had fewer days under ventilation (3.91 ± 6.2 vs. 3.15 ± 4.6; mean difference, −0.76 (95% CI, −1.10; −0.43); *P* <0.001), and more had 28 ventilator-free days (16.07 ± 12.2 vs. 18.33 ± 11.6; mean difference, 2.30 (95% CI, 1.57; 3.00); *P* <0.001), compared with patients who were mechanically ventilated in the preintervention period. Although patients in the postintervention period stayed fewer days in the ICU, they presented similar hospital lengths of stay compared with patients in the preintervention period. The ICU and in-hospital mortality rates were lower in the postintervention period (36.8% (95% CI, 35.0; 38.8) vs. 29.6% (95% CI, 27.8; 31.4), *P* <0.001; and 45.4% (95% CI, 43.5; 47.4) vs. 38.1% (95% CI, 36.2; 40.1), *P* <0.001, respectively) (Table 4).

**Table 4:** **Characteristics of the mechanically ventilated patients** ^**a**^

	**Preintervention (** ***n*** **= 2,446)**	**Postintervention (** ***n*** **= 2,405)**	***P*** **-value**
**Characteristics**	**October 2010 to September 2011**	**October 2011 to October 2012**	
Age, yr, mean ± SD	62.37 ± 19	61.99 ± 18	0.467
Male, *n* (%)	1,306 (53.4)	1,275 (53.0)	0.792
BMI, kg/m^2^, mean ± SD	26.7 ± 6	26.7 ± 6	0.726
SAPS III score, mean ± SD	52.65 ± 19	51.44 ± 20	0.031
Charlson index score, median (IQR)	1 (0; 2)	1 (0; 2)	0.181
Admission type, *n* (%)			0.119
Medical	1,547 (63.2)	1,452 (60.4)	
Elective surgical	767 (31.4)	811 (33.7)	
Emergency surgical	132 (5.4)	142 (5.9)	
Diagnosis category, *n* (%)			
Cardiovascular	219 (9.0)	208 (8.6)	0.708
Sepsis	505 (20.6)	548 (22.8)	0.071
Neurological	284 (11.6)	276 (11.5)	0.883
Respiratory^b^	303 (12.4)	182 (7.6)	<0.001
Orthopedic surgery	28 (1.1)	31 (1.3)	0.647
Neurosurgery	63 (2.6)	47 (2.0)	0.146
Thoracic surgery	24 (1.0)	31 (1.3)	0.311
Cardiac surgery	545 (22.3)	627 (26.1)	0.002
Resources during ICU, *n* (%)			
RRT	174 (7.1)	185 (7.7)	0.441
Vasopressors	1,321 (54)	1,436 (59.7)	<0.001
Noninvasive ventilation	639 (26.1)	612 (25.4)	0.590
Mechanical ventilation, *n* (%)			
During ICU stay	2,446 (100)	2,405 (100)	–
At ICU admission	1,677 (68.6)	1,673 (69.6)	0.450
Tracheotomy	314 (12.8)	237 (9.9)	0.001
Length of mechanical ventilation			
Mean ± SD	3.91 ± 6.2	3.15 ± 4.6	<0.001
Median (IQR)	1 (1; 4)	1 (1; 3)	0.001
MV >24 hr	1,007 (41.1)	971 (40.2)	0.510
28 ventilator-free days			
Mean ± SD	16.07 ± 12.2	18.33 ± 11.6	<0.001
Median (IQR)	24 (0; 27)	26 (0; 27)	<0.001
ICU LOS, days			
Mean ± SD	8.87 ± 11.9	7.46 ± 9.2	<0.001
Median (IQR)	5 (2; 12)	4 (2; 10)	<0.001
Total hospital LOS, days			
Mean ± SD	23.75 ± 33.7	23.80 ± 31.3	0.961
Median (IQR)	13 (6; 28)	13 (6; 27)	0.707
Mortality, % (95% CI)			
ICU mortality	36.8% (35.0; 38.8)	29.6% (27.8; 31.4)	<0.001
Hospital mortality	45.4% (43.5; 47.4)	38.1% (36.2; 40.1)	<0.001

^a^BMI, Body mass index; ICU, Intensive care unit; MV, Mechanical ventilation; RRT, Renal replacement therapy. ^b^Respiratory diagnosis except pneumonia.

**Figure 2:**
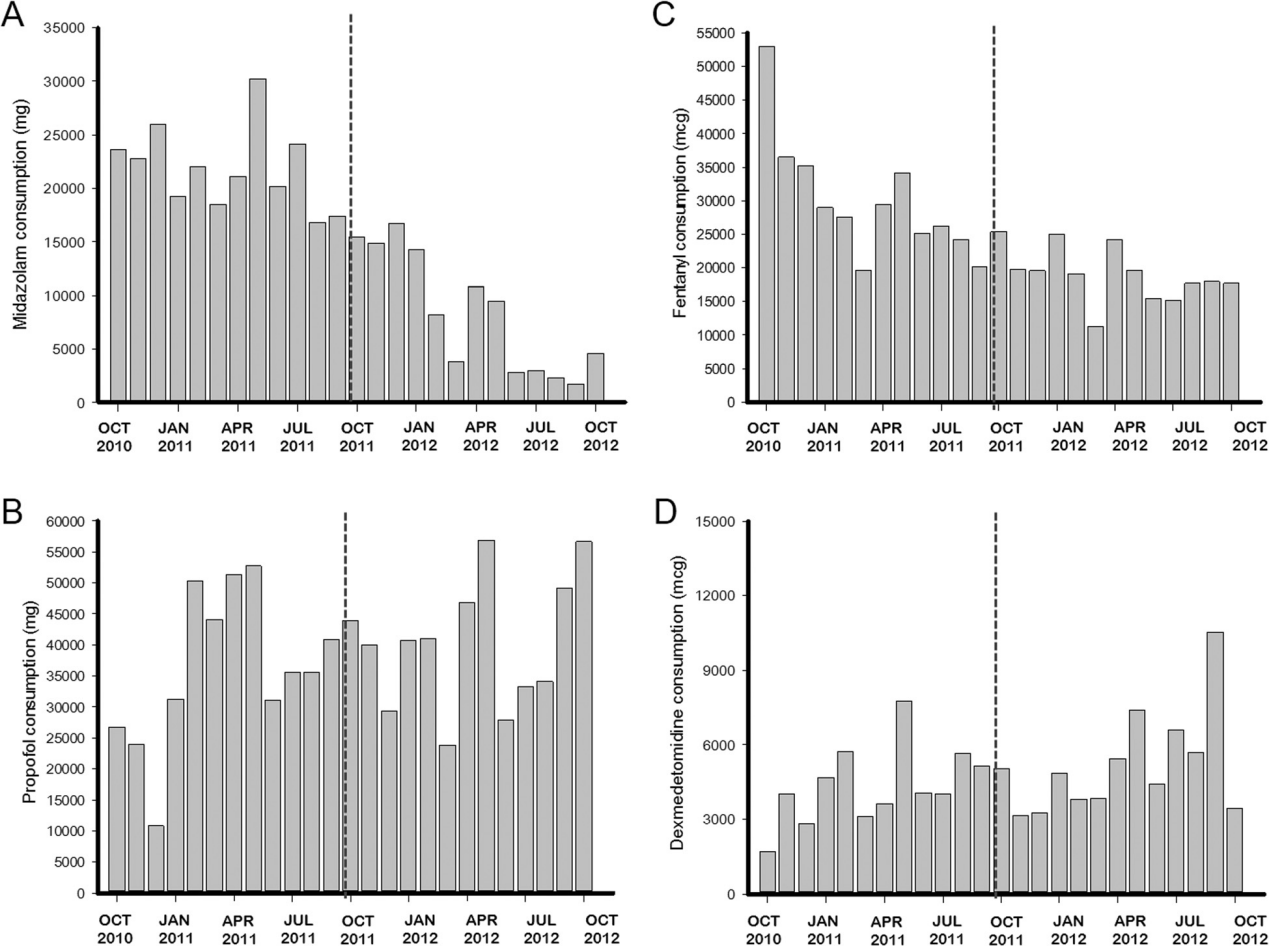
**Monthly consumption in mg of sedoanalgesic medications. (A)** Midazolam. **(B)** Propofol. **(C)** Fentanyl. **(D)** Dexmedetomidine.

The use of midazolam presented the largest decrease between both periods. Monthly midazolam consumption in milligrams per ICU decreased from 21,825 ± 3,835 to 8,297 ± 5,628 (mean difference, −13,528 (95% CI, −17,548; −9,508), *P* <0.001). The results were similar after the correction for MV duration (329 ± 70 vs. 163 ± 115; mean difference, −167 (95% CI, −246; −87), *P* <0.001). In contrast to midazolam, the average consumption per day of MV for propofol (*P* = 0.007), dexmedetomidine (*P* = 0.017) and haloperidol (*P* = 0.002) increased in the postintervention period, with no change observed in fentanyl consumption (*P* = 0.120) (Table 5, Figure 2 and Additional file 1: Figure S3).

**Table 5:** **Sedative consumption and differences between periods** ^**a**^

	**Preintervention**	**Postintervention**		
	**October 2010 to September 2011**	**October 2011 to October 2012**	**Mean difference (95% CI)**	***P*** **-value**
Monthly average midazolam consumption (mg)	21,825 ± 3,835	8,297 ± 5,628	−13,528 (−17,548; −9,508)	<0.001
Average midazolam consumption per day of MV (mg/day)	329 ± 70	163 ± 115	−167 (−246; −87)	<0.001
Monthly average propofol consumption (mg)	36,210 ± 12,506	40,297 ± 10,459	4,088 (−5,422; 13,598)	0.383
Average propofol consumption per day of MV (mg/day)	541 ± 190	779 ± 210	238 (72; 404)	0.007
Monthly average fentanyl consumption (μg)	30,021 ± 9,029	19,057 ± 4,302	−10,964 (−16,670; −5,258)	0.001
Average fentanyl consumption per day of MV (μg/day)	458 ± 170	370 ± 90	−88 (−202; 25)	0.120
Monthly average dexmedetomidine consumption (μg)	4,350 ± 1,592	5,186 ± 2,066	836 (−700; 2,372)	0.272
Average dexmedetomidine consumption per day of MV (μg/day)	65 ± 20	100 ± 40	35 (07; 62)	0.017
Monthly average haloperidol consumption (mg)	176.4 ± 24	195.0 ± 61	18.7 (−20.0; 57.5)	0.328
Average haloperidol consumption per day of MV (mg/day)	03 ± 0.5	04 ± 1.0	1.1 (0.4; 1.7)	0.002

^a^MV, Mechanical ventilation.

### Comparison of length of mechanical ventilation and midazolam consumption among units

**Figure 3:**
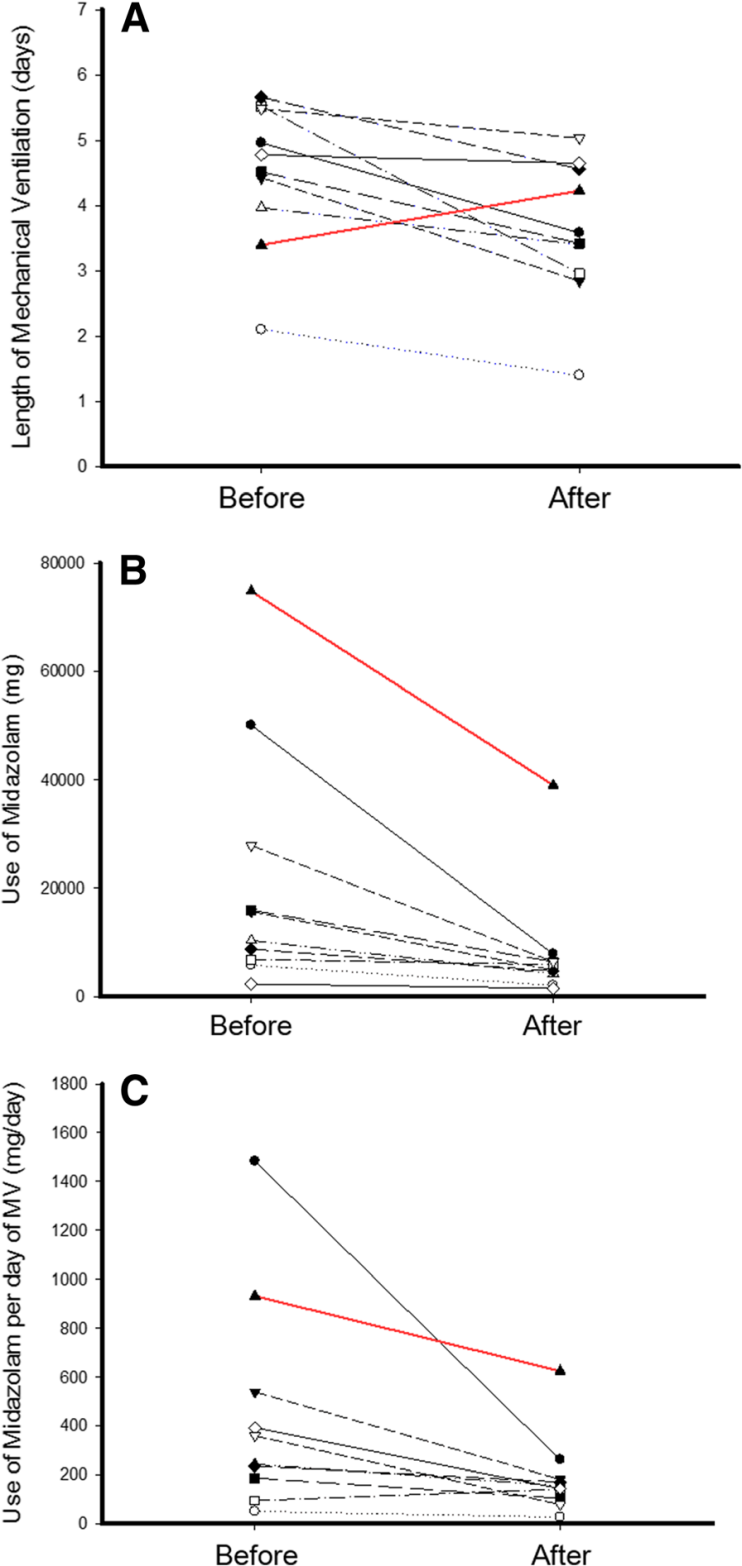
**Comparisons of length of mechanical ventilation and midazolam consumption among units in each of the ten units in the quality improvement project. (A)** Length of mechanical ventilation. **(B)** Midazolam consumption. **(C)** Midazolam consumption per day of mechanical ventilation (MV). The *red line* denotes the only unit which showed an inclination toward increased length of mechanical ventilation in the period (mean difference, 0.83 (95% CI, −1.10; 2.76), *P* = 0.38).

The mean length of MV decreased across the ten units, except for one during the before and after periods (Figure 3). The highest decrease was from 5.52 ± 3.1 vs. 2.95 ± 0.9 days (mean difference, −2.57 (95% CI, −4.56; −0.59), *P* = 0.015 (unit 6)). There was only one unit with an inclination toward an increase in the length of MV, which was from 3.39 ± 1.9 vs. 4.22 ± 2.7 days (mean difference, 0.83 (95% CI, −1.10; 2.76), *P* = 0.38 (unit 9)) (Figure 3, red line). Regarding crude and adjusted midazolam consumption, midazolam consumption decreased in all units in the postintervention period compared with the preintervention period (Figure 3).

### Interrupted time series analysis

#### Patients’ characteristics

The analyses of age and SAPS III did not show any trend during the observed period. Both models for age and SAPS III demonstrated no signal (full model, *P* = 0.574 and *P* = 0.176, respectively) for the overall cohort and similar results for MV patients (full model, *P* = 0.308 and *P* = 0.462, respectively). The crude data for age and SAPS III over time are shown in Additional file 1: Figures S1 and S2. The use of MV (*P* = 0.621) among admitted patients and the hospital mortality rate (*P* = 0.204) over time did not exhibit any trend during the period (Additional file 1: Figure S4).

#### Length of mechanical ventilation

**Figure 4:**
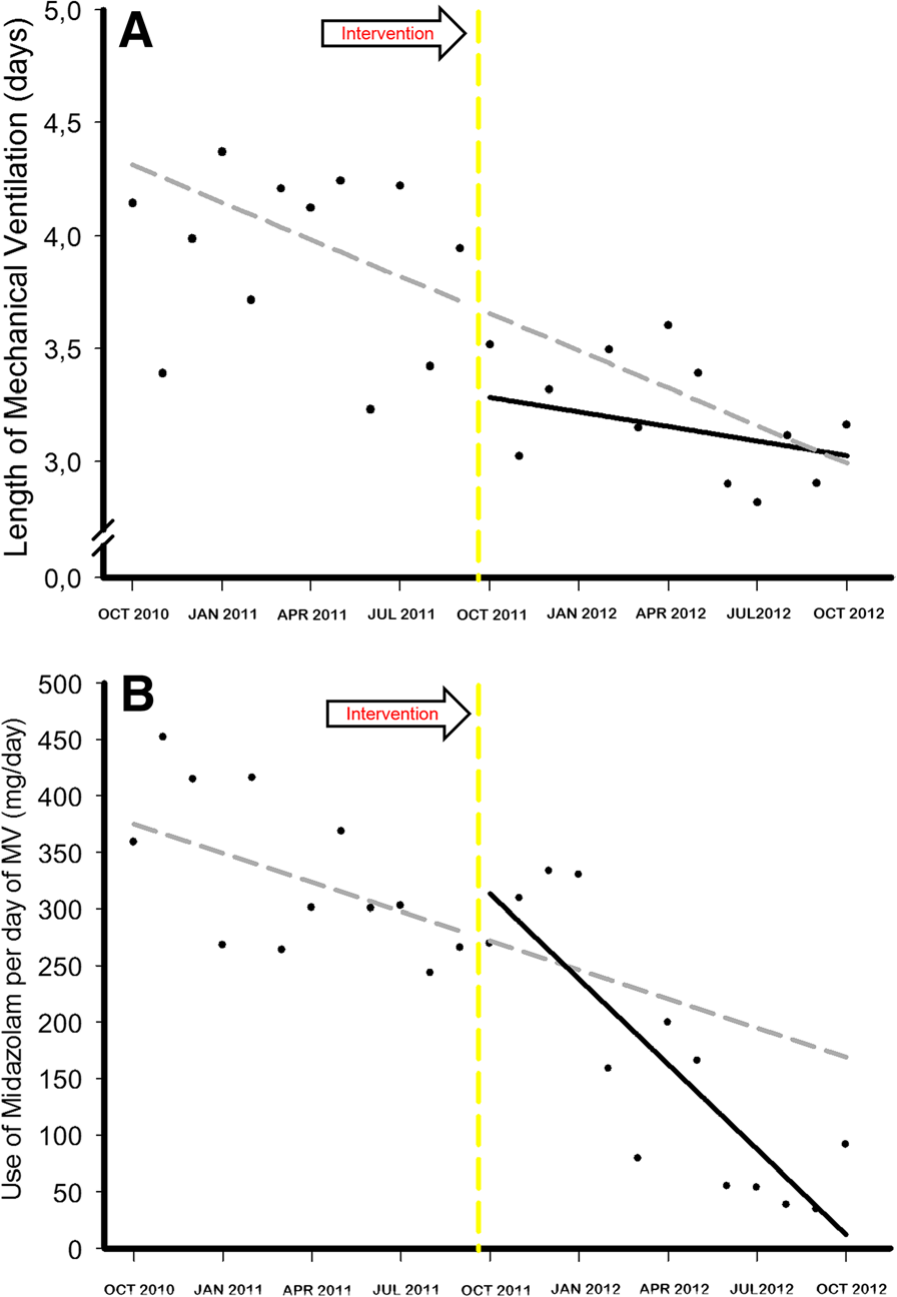
**Interrupted time series from the autoregressive integrated moving average model.** Length of mechanical ventilation (MV) **(A)** and adjusted midazolam consumption **(B)** over time. *Solid black circles* represent the average data per month. *Solid black line* represents the fitted line for the observed data after the protocol implementation. *Gray dashed line* represents the forecasted values from the model if the protocol was not implemented during the period. *Yellow dashed line* represents when the intervention began.

**Figure 5:**
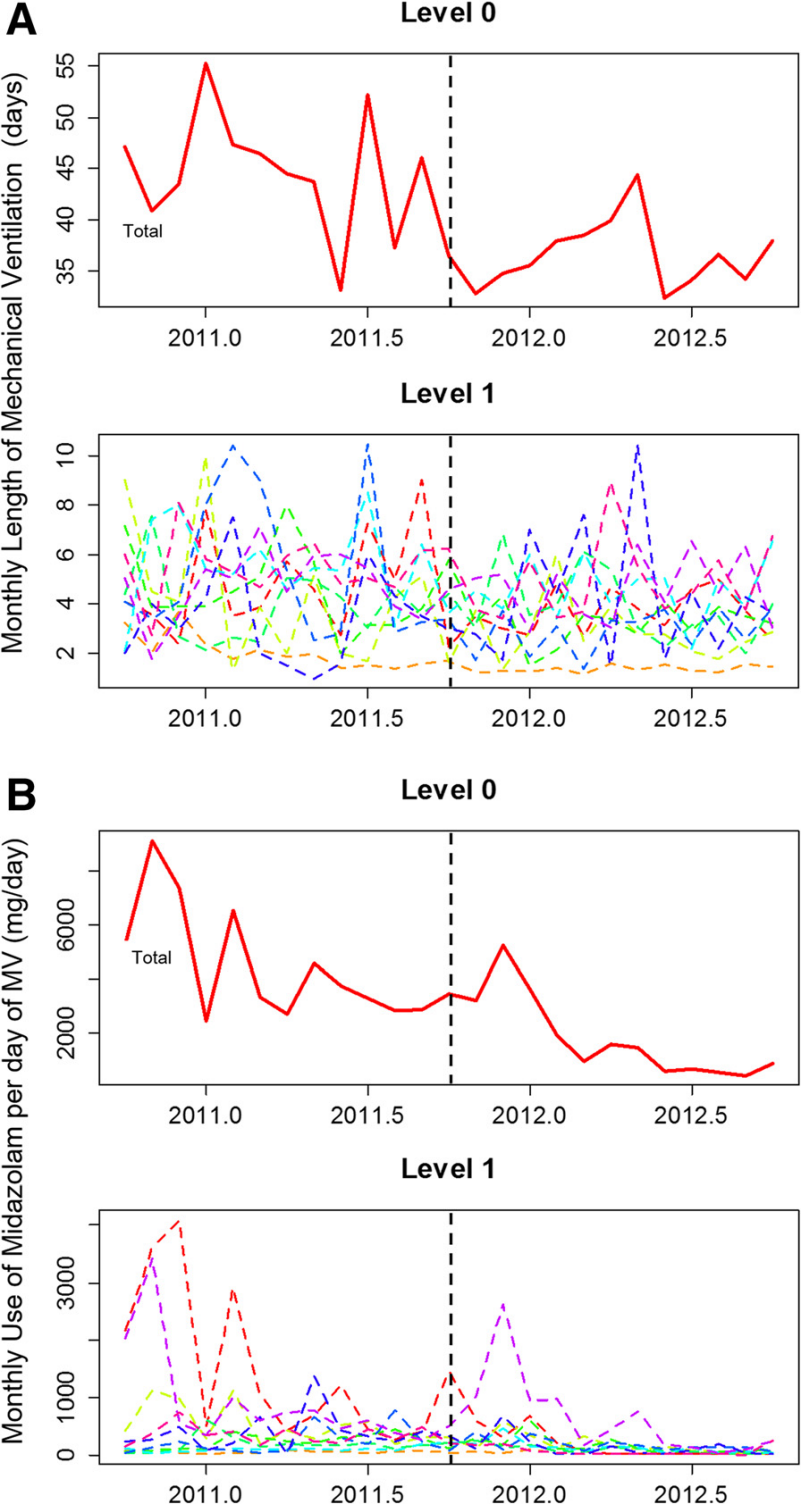
**Results from the hierarchical time series model.** Length of mechanical ventilation (MV) **(A)** and adjusted midazolam consumption **(B)** over time. Level 0 denotes the modeled time series using a bottom-up method for the entire network. Level 1 denotes the independent time series for each of the ten units analyzed. *Dashed black lines* represent when the intervention began.

In analyzing our primary endpoint, we found that there was a secular trend associated with a natural decline of the time under MV (secular trend β_1_ = −0.055, *P* = 0.012). We also observed an immediate effect of the intervention (intervention β_2_ = −0.976, *P* <0.001) toward decreased length of MV. However, the trend (slope) in the postintervention period remained unchanged (postintervention trend β_3_ = 0.039, *P* = 0.095) (Table 6, Figure 4A and Additional file 1: Figure S5). Regarding the hierarchical time series analysis, it is important to note that the majority of units presented a similar and synchronized response to the intervention (crude time series, Figure 5A, level 1). Furthermore, the impact of the intervention could be reproduced by the hierarchical times series analysis (Table 6 and Figure 5A).

**Table 6:** **Interrupted time series analysis for length of mechanical ventilation and midazolam consumption** ^**a**^

	**Average crude data**						**Modeled data from hierarchical time series**					
	**Length of MV (days)**			**Midazolam consumption (mg/day of MV)**			**Length of MV (days)**			**Midazolam consumption (mg/day of MV)**		
	**β**	**SE**	***P*** **-value**	**β**	**SE**	***P*** **-value**	**β**	**SE**	***P*** **-value**	**β**	**SE**	***P*** **-value**
Constant (β_0_)	4.369	0.169	<0.001	383.5	30.3	<0.001	52.608	3.426	<0.001	5406.7	488.7	<0.001
Secular trend (β_1_)	−0.055	0.019	0.012	−8.585	3.77	0.038	−1.011	0.396	0.023	−199.4	60.8	0.005
Intervention (β_2_)	−0.976	0.067	<0.001	−0.045	1.017	0.97	−0.979	0.158	<0.001	−0.431	0.926	0.65
Postintervention trend (β_3_)	0.039	0.022	0.095	−19.457	5.93	0.005	1.080	0.545	0.068	−183.9	74.3	0.026

^a^MV, Mechanical ventilation.

The results for individual-level patient length of MV from the mixed model, which allowed adjustments for patient levels (SAPS III, Charlson score and vasoactive drugs) and unit levels (random component) showed similar results, despite the postintervention period. The secular trend was associated with a natural decline in the length of MV (secular trend β_1_ = −0.016, *P* <0.001). We also observed an immediate effect of the intervention (intervention β_2_ = −0.078, *P* = 0.020) toward decreasing length of MV and a significant positive slope in the postintervention period (postintervention trend β_3_ = 0.012, *P* = 0.011). The mixed model presented a better overall model fit when compared to a similar model without random components (*P* <0.001 by likelihood ratio test). We found that the random effects explained almost 10% of the mixed model variance. The random intercepts and their errors around each estimate are described Additional file 1: Figure S7. The figure reinforces that the variance explained by each unit is minor, because the majority of units are near the zero value.

#### Use of midazolam

There was already an effect in the secular trend (secular trend β_1_ = −8.585, p = 0.038) associated with a natural decrease in the use of midazolam per day of MV and no effect immediately after the intervention (intervention β_2_ = −0.045, *P* = 0.97). However, there was a clear significant effect of the QI project in the postintervention period (postintervention trend β_3_ = −1.946, *P* = 0.005), changing the slope of the natural decline in the rate of midazolam consumption (Table 6 and Figure 4B). Our sensitivity analysis using the hierarchical time series model showed similar results (Table 6 and Figure 5B). The hierarchical time series from the crude midazolam data consumption is shown in Additional file 1: Figure S6. The impact of the intervention also seemed to be simultaneous across the majority of the units (Figure 5B and Additional file 1: Figure S6, Level 1).

#### Midazolam consumption vs. length of mechanical ventilation

**Figure 6:**
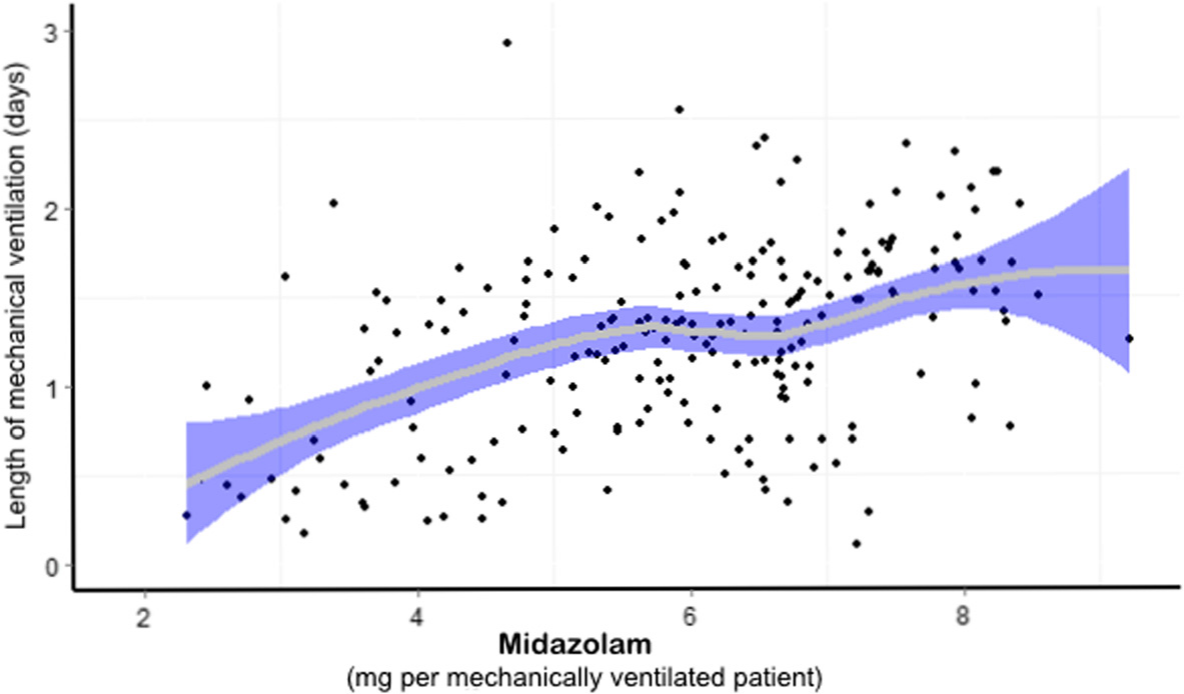
**Association between midazolam consumption and length of mechanical ventilation.** A positive, nonlinear association between midazolam and length of mechanical ventilation was found (*P* = 0.022) in a mixed linear model. Each point represent the variable per each intensive care unit per month. *x*- and *y*-axes are in natural logarithmic scale. *Solid gray line* represents the best fit between variables. *Blue bands* represent the 95% of confidence interval from the fit.

We found a positive, nonlinear association between midazolam consumption and length of MV (*P* = 0.022), adjusted for SAPS III score and taking account the effect of repeated and clustered data (Figure 6).

#### Other sedatives

For propofol and fentanyl, we did not observe any effect in secular trends during the observation period. The consumption of haloperidol showed no changes in the secular trend or postintervention; however, the consumption of haloperidol significantly increased immediately after intervention (intervention β_2_ = 0.869, *P* <0.001). The consumption of dexmedetomidine showed no changes in the secular trend or immediately after intervention; however, the consumption of dexmedetomidine increased in the postintervention period (postintervention trend β_3_ = 0.129, *P* = 0.046).

## Discussion

In this multicenter QI project, we found that implementation of a minimal sedation policy in a group of nonteaching hospitals is feasible and that systematic monitoring of sedative consumption seems to be a useful instrument for supporting the accomplishment of a protocol on a large scale. Additionally, we could reproduce the clinical benefits that have been previously demonstrated in controlled settings.

Interestingly, the average consumption of midazolam decreased its slope following the campaign, but not immediately after the intervention. In contrast, the duration of MV changed importantly near the implementation, changing its level (negative coefficient for β_2_). This effect was maintained over a 1-year period, without reversing, although the positive slope after the intervention represented a waning trend for the initial effect of the campaign [31]. This finding could be attributable to the higher effect of our intervention in situations of oversedation, which should be less common following the campaign. Furthermore, we speculate that the important decrease in the duration of MV near the beginning of the intervention might be secondary to the implementation of daily sedation interruption practice (not systematically measured in our study), which is reflected in the pattern observed for the use of neuroleptic agents. Although the focus of the protocol was not exclusively on the aim of decreasing benzodiazepines, their use was greatly affected by the campaign. This finding is possibly based on the fact that midazolam was the main sedative drug used in our units in the baseline period and on current knowledge regarding the association between benzodiazepines and poor outcomes [4,12]. The positive association between midazolam consumption and time under MV reinforces the link to sedative use as a monitoring tool for quality of sedation. Moreover, it is possible that the decrease in midazolam use was responsible for a part of the reduction in length of MV, aside from the QI project. Nevertheless, it is not possible to establish a causal association between these variables.

Pre–post studies are naturally predisposed to important changes in patient’s characteristics and natural improvement over time. To overcome this barrier, we applied an ITS analysis, which is the strongest quasi-experimental design for evaluating the effects of time-delimited interventions [32,33]. Furthermore, we fit a multilevel sensitivity analysis to account for patient and unit characteristics, correctly addressing repeated and correlated data. In a crude analysis, our population changed slightly and presented low mortality rates in the postintervention period (Tables 3 and 4). However, crude analysis is underpowered to address the question properly, because the confounding due to secular trends is not taken into account. Indeed, the results from time series analysis demonstrated that important features of the population remained stable during the observed period (age, SAPS III, use of MV and in-hospital mortality), which ensures general stability of severity during the observational period and enhances our results.

The implementation of new routines of care has been studied extensively, and current knowledge indicates that the use of isolated traditional teaching tools, such as formal classes or the distribution of detailed written protocols, are consistently inefficacious [22,28,40,41]. In contrast, the multifaceted intervention presented better results, applying continuous education, opinion leader development, reminders and permanent audit and feedback [27,28,30,31,41,42]. We believe that our positive results are attributable to the proper use of these “new” tools in a multifaceted approach, which involved a multidisciplinary team. Indeed, nurses, clinical pharmacists and respiratory therapists were invited to participate in the development of local guidelines, as well as in the meetings and conferences. To ensure the involvement of the entire team, each unit had daily multidisciplinary rounds, which included a checklist concerning sedoanalgesia status for each patient and opportunities for the staff to participate actively in decision-making processes. Another important point to take from our QI project is the use of a simple and objective instrument for monitoring compliance relative to a task. We considered that the traditional measurement of an indicator of process, such as adherence to down-titration of sedatives, may be prone to several judgment biases and may not represent the ideal approach. Using sedative consumption per ICU per month, the coordinator could objectively measure compliance with light sedation policy, provide accurate feedback and perform benchmarking among the different units.

The primary strength of our study is the large number of patients involved and the use of reliable and accurate data. Additionally, because it was a multicenter QI project, the study has a chance of good external validity, in which the implementation of this policy and its benefit do not seem to be personnel-dependent. Indeed, the multilevel analysis raised insights that, for a range of units, the effect of the campaign was homogeneous. In fact, the unit-level response for the majority of units was uniform, and, although the random component introduced by multilevel analysis was significant, only 10% of the total variance in length under MV was accounted for by the clustering per each unit. We demonstrate that monitoring sedative consumption can be a useful tool for protocol implementation management on a large scale, mainly focusing on benzodiazepines, which was recently reported in another single-center study [43].

Nevertheless, the present study also has some weaknesses. Pre–post analyses are always prone to the concurrent effects of simultaneous initiatives. We tried to avoid such problems by employing a strict methodology that takes into consideration the secular trend, such as the ITS analysis through ARIMA models [32]. We did not evaluate individual patient consumption of sedatives; thus, all of our inferences regarding this information were derived from the ICU perspective, and we could not calculate true measurements of dispersion taking into consideration the individual patient unit of analysis. Accordingly, we cannot affirm that the reduction of sedative use was completely attributable to its reduction in mechanically ventilated patients, because these drugs can be used in spontaneously breathing patients as well. On the basis of our experience at the bedside, we are convinced that this last category of use is quantitatively less important and should have been kept constant during the study period, because the most important clinical characteristics remained the same. Although the HMO in Sao Paulo did not show any service change during the observed period and the referees and specialties available in the hospitals were maintained constant, we cannot guarantee that some case mix variation could have exerted influence in our analysis. Regarding the statistical plan, nonlinear methods could be an alternative to time series; however, our time series did not have enough data points for a reliable analysis. We did not systematically evaluate important markers of sedation policy, such as delirium incidence and nursing workload. In addition, we did not evaluate other sedoanalgesic drugs used in the study period.

The implications of our results are clear. In scenarios similar to ours in terms of sedative consumption and MV duration, we can affirm that the implementation of a simple sedation policy using a monitoring tool is feasible and provides clinical benefits. Considering the low incremental costs, we strongly suggest that this initiative is cost-effective, and most likely cost-saving as well, from the societal perspective. Our initiative was performed with continuous attention focused on keeping it sustainable concerning the amount of resources employed. We aimed to ensure that all of our tools (from teaching to monitoring) would facilitate the comprehension and implementation of the sedation policy and that complicated algorithms would be avoided. A laborious bedside audit process was replaced by a measurement of medication consumption that could still be used in feedback sessions with the leaders of each ICU. Considering a multicenter QI project, the local variation at each unit exerted an important role in the management and results. Indeed, a unit with a low volume of mechanically ventilated patients (3% to 5% per year) showed a wide variability in length of MV and sedative consumption per month, which did not allow us to draw inferences and make determinations of clear barriers to implementation. One other unit with a low number of beds challenged our project, probably because general guidelines are less applicable to customized settings.

## Conclusion

The implementation of a light sedation policy is feasible in a group of nonteaching hospitals, and systematic monitoring of sedative consumption seems to be a simple and objective instrument for supporting the accomplishment of a protocol on a large scale. Furthermore, this QI project reproduced the clinical benefits that have previously been demonstrated in controlled settings.

## Key messages

- Currently, there is a gap between real-life practice and guidelines regarding sedation practice in ICUs. There is a tendency toward oversedation, which is associated with worse outcomes.
- A multifaceted implementation approach improved the practice of light sedation in a network of nonteaching hospitals.
- Sedative consumption seems to be a guiding tool to help managers to tailor a campaign and seems to be feasible in a large-scale program.
- Decreases in benzodiazepine consumption are associated with shorter length of mechanical ventilation.

## Additional file

Additional file 1:
**Additional statistical analysis plan and additional results.**

## Abbreviations

ARIMA: Autoregressive integrated moving average
CRF: Case report formulary
HMO: Health maintenance organization
ICU: Intensive care unit
IQR: Interquartile range
ITS: Interrupted time series
MV: Mechanical ventilation
QI: Quality improvement program
SAPS III: Simplified Acute Physiology Score III
VAP: Ventilator-associated pneumonia
