# Supplementary material for: Evaluation of a minimal sedation protocol using ICU sedative consumption as a monitoring tool: a quality improvement multicenter project
Source: Crit Care. 2014 Oct 24;18(5):580. doi: 10.1186/s13054-014-0580-3 (PMC4234844; doi:10.1186/s13054-014-0580-3)
Supplement: Additional file 1: — Additional statistical analysis plan and additional results. [file 13054_2014_580_MOESM1_ESM.pdf]

Supplemental Digital Content

Evaluation of a minimal sedation protocol using ICU sedative consumption as a monitoring tool: a quality improvement multicenter project - On behalf of the AMIL Critical Care Group

Otavio T. Ranzani, Evelyn Senna Simpson, Talita Barbosa Augusto, Sylas Bezerra Cappi, Danilo Teixeira Noritomi

Data analysis

To analyze the effect of the campaign, we planned a quasi-experimental design using interrupted time series (ITS) in order to control for secular trends [1-4]. In an ITS design, data are collected at multiple instances over time before and after an intervention (interruption) and is the strongest, quasi-experimental design to evaluate effects of time-delimited interventions. An advantage of ITS design is that it allows investigation of potential biases further them the secular trend, as duration of the intervention (i.e. the intervention might have an effect for the first two months only and not a sustained one).

Interrupted time series analysis and ARIMA models

To investigate such biases, we run autoregressive integrated moving average models (ARIMA). Two parameters define each segment (period before and after the intervention) of a time series: level and trend. The level is the value of the series at the beginning of a given time interval. The trend is the rate of change of a measure (slope) during a segment. To examine the results, we might analyze if there are changes in level and trend that follow an intervention. In general, a change in level constitutes an abrupt intervention effect and a change in trend represents a gradual change in the value of the outcome. This model involves a multilinear regression and we can specify the following linear regression model to estimate the level and trend for our primary endpoint – length of mechanical ventilation:

$$Y_t = \beta_0 + \beta_1 * \text{time} + \beta_2 * \text{intervention}_t + \beta_3 * \text{time after intervention}_t + e_t$$

Here, Y_t is the mean number of days of mechanical ventilation per patient in month t; time is a continuous variable indicating time in months at time t from the start of the observation period; intervention is an indicator for time t occurring before (intervation = 0) or after (intervention = 1) the campaign, which was implemented at month 13 in the series; and time after intervention is a continuous variable counting the number of months after the intervention at time t, coded 0 before the campaign and (time minus 12) after the campaign. So, β_0 estimates the baseline level of the outcome at time zero; β_1 estimates the level change in the mean length of mechanical ventilation before the campaign (baseline trend). β_2 estimates the level change in the mean monthly length of mechanical ventilation per patient immediately after the intervention and β_3 estimates the change in the trend in the mean monthly length of mechanical ventilation per patient after the campaign. The error term e_t at time t represents the random variability not explained by the model. The above equation is the linear component of the model. The full model can be represented as:

$$Y_t = \beta_0 + \beta_1 * \text{time} + \beta_2 * \text{intervention}_t + \beta_3 * \text{time after intervention}_t + e_t, \; ARIMA\ (p,q,d)$$

Here, the ARIMA components are: p - order of the autoregressive part; d - degree of first differencing involved and q - order of the moving average part. In the case of Y_t being days under mechanical ventilation, we have:

$$Y_t = 4.369 + (-0.055 * \text{time}) + (-0.976 * \text{intervention}_t) + (0.039 * \text{time after intervention}_t) + e_t,\ ARIMA\ (2,0,1)$$

The ARIMA (2,0,1) is represented in this case by:

$$Y_t = 4.369 + (-0.055 * \text{time}) + (-0.976 * \text{intervention}_t) + (0.039 * \text{time after intervention}_t) +$$

$$e_t + (-1.295 * Y_{t-1}) + (-0.296 * Y_{t-2}) + (-0.986 * e_{t-1})$$

where e_t in this case is the white noise with standard deviation 0.235.

Below is the hypothetical data to exemplify how to organize the data to conduct an interrupted time series analysis. In this short example, the intervention occurred after 5 months of the observed period. Time can be seconds, hours, days, months, and years; however it must be at regular intervals for this classical analysis.

Secular time	Intervention	Time after intervention	Days under mechanical ventilation
1	0	0	10,3
2	0	0	11,0
3	0	0	7,7
4	0	0	8,9
5	1	1	6,4
6	1	2	6,0
7	1	3	5,5
8	1	4	4,5
9	1	5	4,1

The assumptions required for ARIMA models, the stationary process which implies that the mean and variance do not change over time, were checked using Phillips-Perron, the Kwiatkowski-Phillips-Schmidt-Shin (KPSS) and Augmented Dickey-Fuller tests. The autocorrelation was checked by visual inspection of autocorrelograms and partial autocorrelograms of the series and its residuals. The White neural network test was used to test for neglected nonlinearity. We also checked for seasonal or cyclical effects by decomposing our series and any of these patterns were detected. The Ljung-Box Q test was run to evaluate a lack of fit of the final ARIMA model.

Hierarchical time series

To fit the hierarchical time series [5] through a bottom-up method, first we get independent time series at the bottom level of the hierarchy (each ICU, Level 1) and then aggregating the independent time series upwards to produce a revised time series for the whole hierarchy. This method accounts for noise and variability between time series and provides additional information in comparison of crude average.

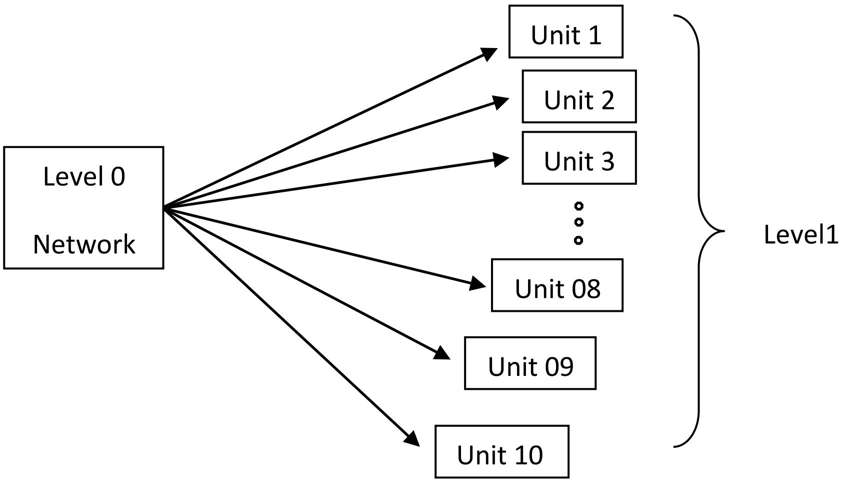

In our study, the Level 0 is the most aggregated level and represents the network level. As depicted in the scheme above, the Level 0 is composed by 10 series at Level 1. The $Y_{0,t}$ is the t_{th} observation of the Level-0 series for t=1,2,3,4,…, 24 (in our study). For Level-1, we denote $Y_{1,t}$ the t_{th} observation of the series at level 1 (Unit level). The total amount of times series is n = 1 + 10 = 11. To represent the aggregation of the observations, the *hts* package used a matrix notation, constructing a n x nk matrix, where nk is the number of time series at the lowest level of the hierarchy.

The hierarchy can be represented by:

$$Y_t = S*y_{k,t}$$

where Yt is a vector of all observations in the hierarchy at time t, S is the summing matrix as defined above, and $y_{K,t}$ is a vector of all observations in the bottom level of the hierarchy at time t.

For forecasting the hierarchical time series, first we generated independent base forecasts for each time series. To take into account the variance and noisy in a hierarchical model, the function *hts* fits a set of revised versions of each forecast which are close to the original ones but according to the hierarchical structure. The equation representing is:

$$Y_h = S*betta_h + e_h$$

Where Y_h is a vector of the h-step-ahead base forecasts for the whole hierarchy, S is the previous defined matrix, $betta_h$ is the unknown mean of the future values of the bottom level and e_h represents the error in the regression and had zero mean and covariance matrix $\sum h$. After fitting the time series for the Level-0, we extracted the values from the *hts* function and

fit an interrupted time series following the previous statements. All the background explicit above and analyses were retrieved through the *hts* package v 4.4 from professor Rob J Hyndman and coworkers [5].

Generalized Linear Mixed Model

To deal with variation in case-mix over time at two levels (patient and unit), we conducted a sensitivity analysis for our main outcome fitting a Generalized Linear Mixed Model through the segmented regression concept, achieving the better fit for correlated responses, in particular for the analysis of our longitudinal and clustered data. Length of MV for each patient was the dependent variable. For the first level (patient level), we adjusted for SAPS-3, Charlson score and vasoactive drugs. For the second level (unit level), we fit random intercepts for each one of 10 units and nested into units variable random components for types and reasons for admission: sepsis syndrome, cardiac surgery and respiratory reason. The model was built with a Poisson distribution with Log as a link function and the covariance structure for the random effects was the AR1 (First-order autoregressive)[34, 35]. In this model, patient-level was constituted by fixed effects. For the unit-level, the factors were fit as random effects. Therefore, we can model the individual differences between units by assuming different random intercepts for each unit.

This analysis was conducted through the *lme4* package and the *glmer* function. The R code for random effects can be defined as (1|*units*), when the 1 requires an intercept and the *units* variable insert a random effect into the model for each unit. We also nested the above variables per *units*, which the code is (1|*units*/sepsis), where factor sepsis is nested in units, for example. To model the within-group correlation not captured by the random effects, we fit the model with a correlation structure as AR1, by the "cor=corAR1" term.

In order to assess the contribution of the random effects on the model we ran two evaluations. One of them was plotting the random intercepts with the error around each estimate. These parameters were retrieved through the *ranef* function on the *lme4* package [8]. Our efigure7 is an example. Another approach was evaluating the variance parameter for random effects, which can represent the percentage of the model explained by the random components [8, 9]. The "interclass correlation coefficient", used for linear mixed models, was adapted and calculated for generalized linear mixed model as described by Goldstein [10]. Therefore, we were able to retrieve the amount of the model attributed to the random effects. To test whether the random components resulted on better overall model fit in comparison to the model without the random component, we ran a Likelihood ratio test [8, 9].

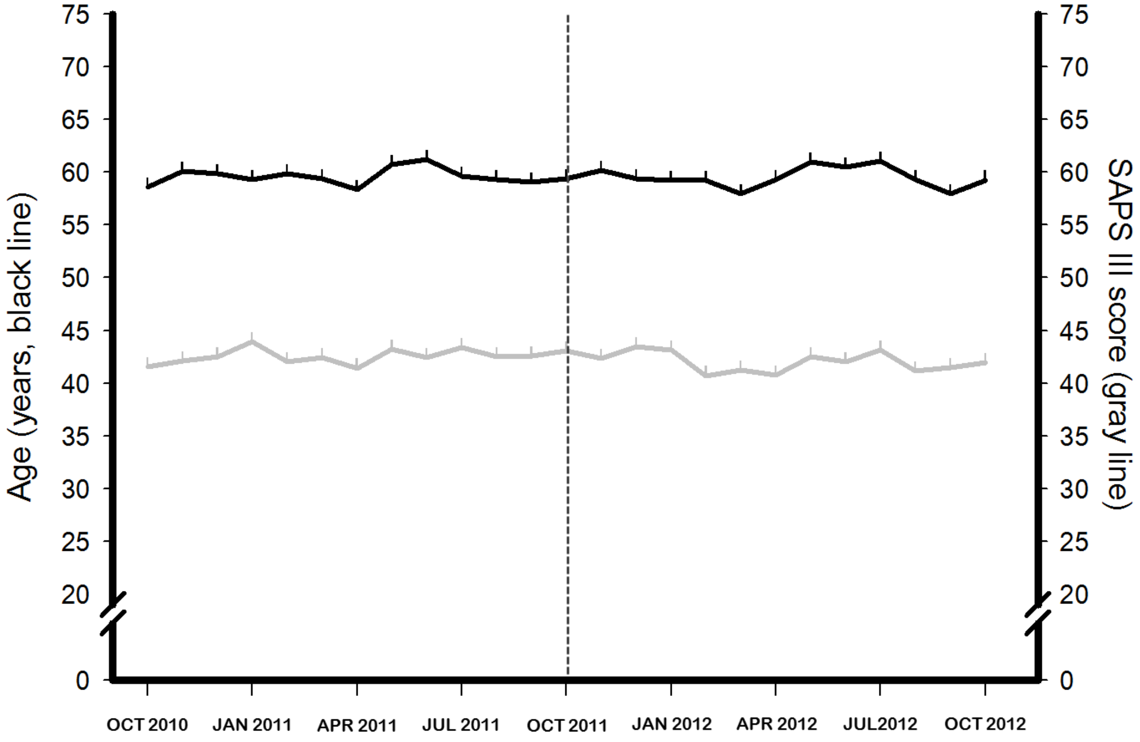

eFigure 1: Crude data about age and SAPS-III during the 2-year period for 22,963 admissions. Time series analysis did not show any trend for age and SAPS-III (full model, p=0.574 and p=0.176, respectively)

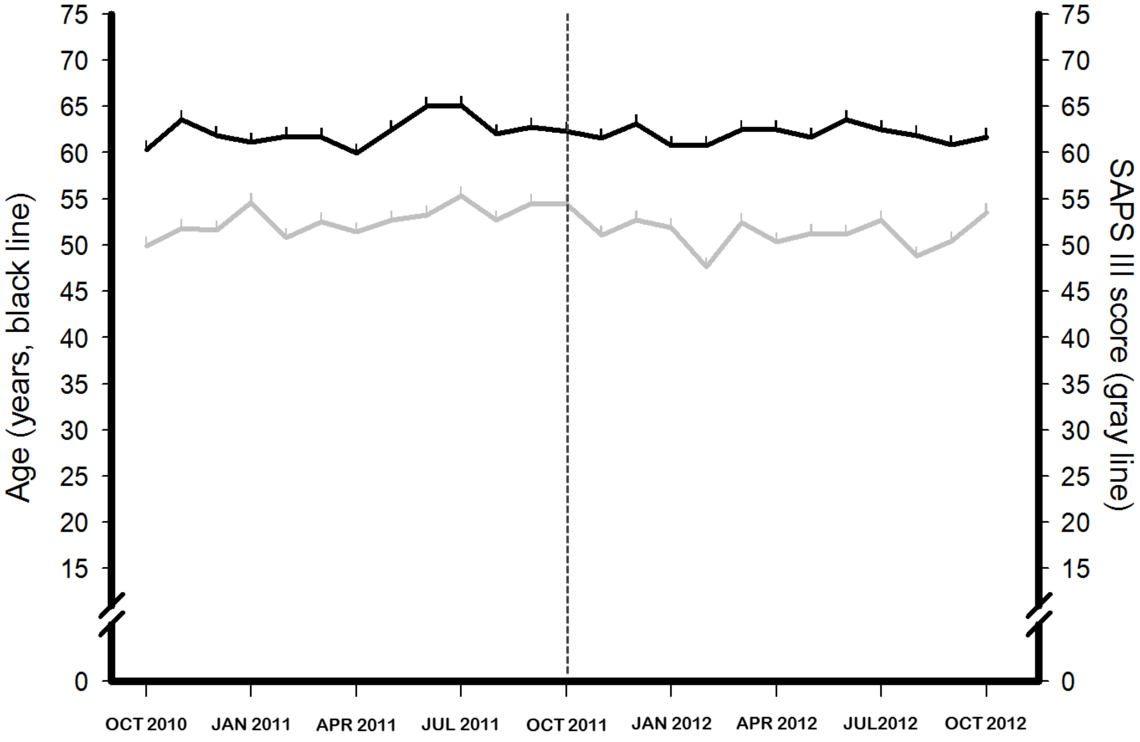

eFigure 2: Crude data about age and SAPS-III during the 2-year period for 4,851 mechanically ventilated patients. Time series analysis did not show any trend for age and SAPS-III patients (full model, p=0.308 and p=0.462, respectively).

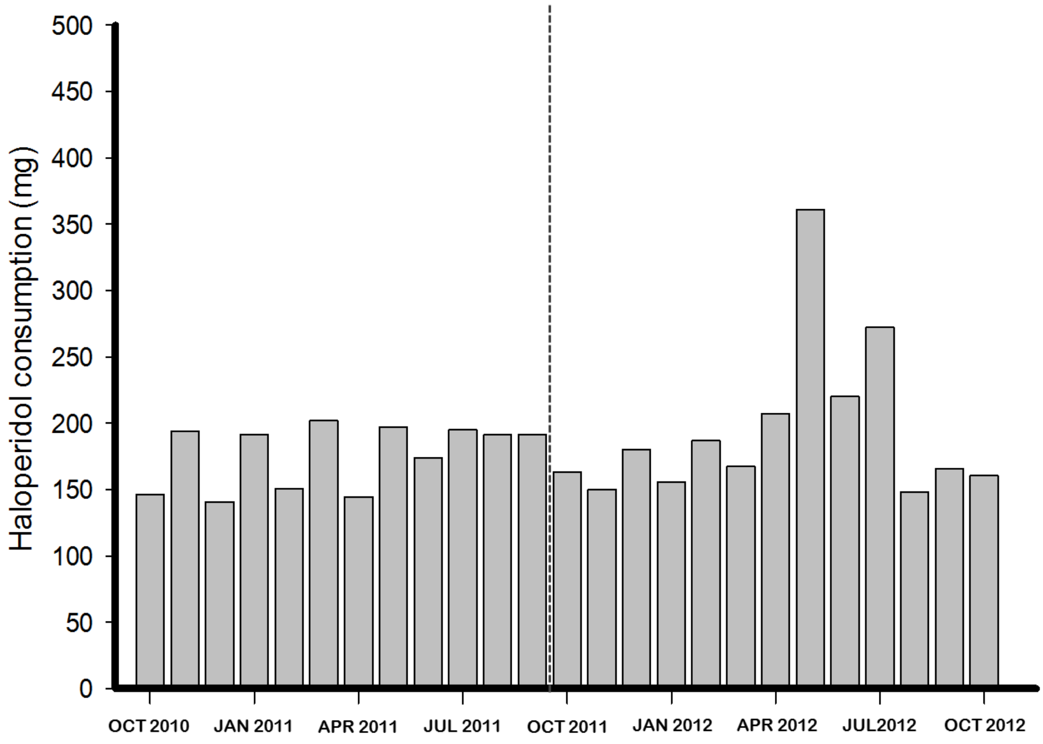

eFigure 3: Crude consumption of haloperidol during the period.

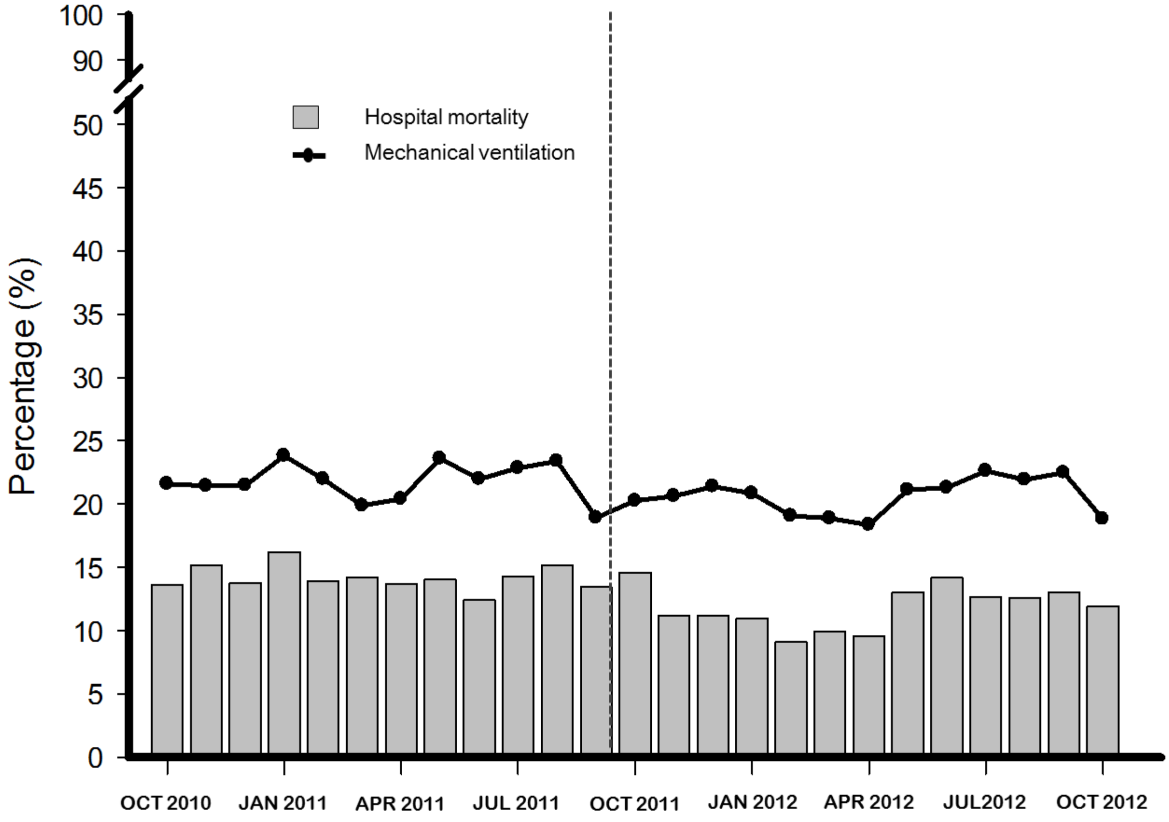

eFigure 4: The mechanical ventilation use and hospital mortality rates over time. There was not any trend during the period.

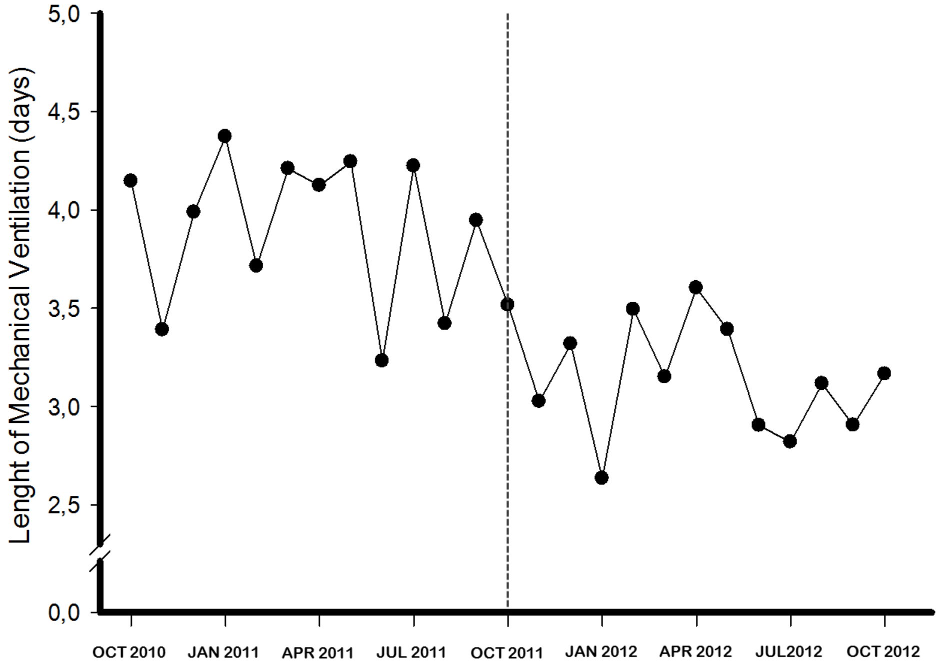

eFigure 5: Crude data for length of mechanical ventilation during the period

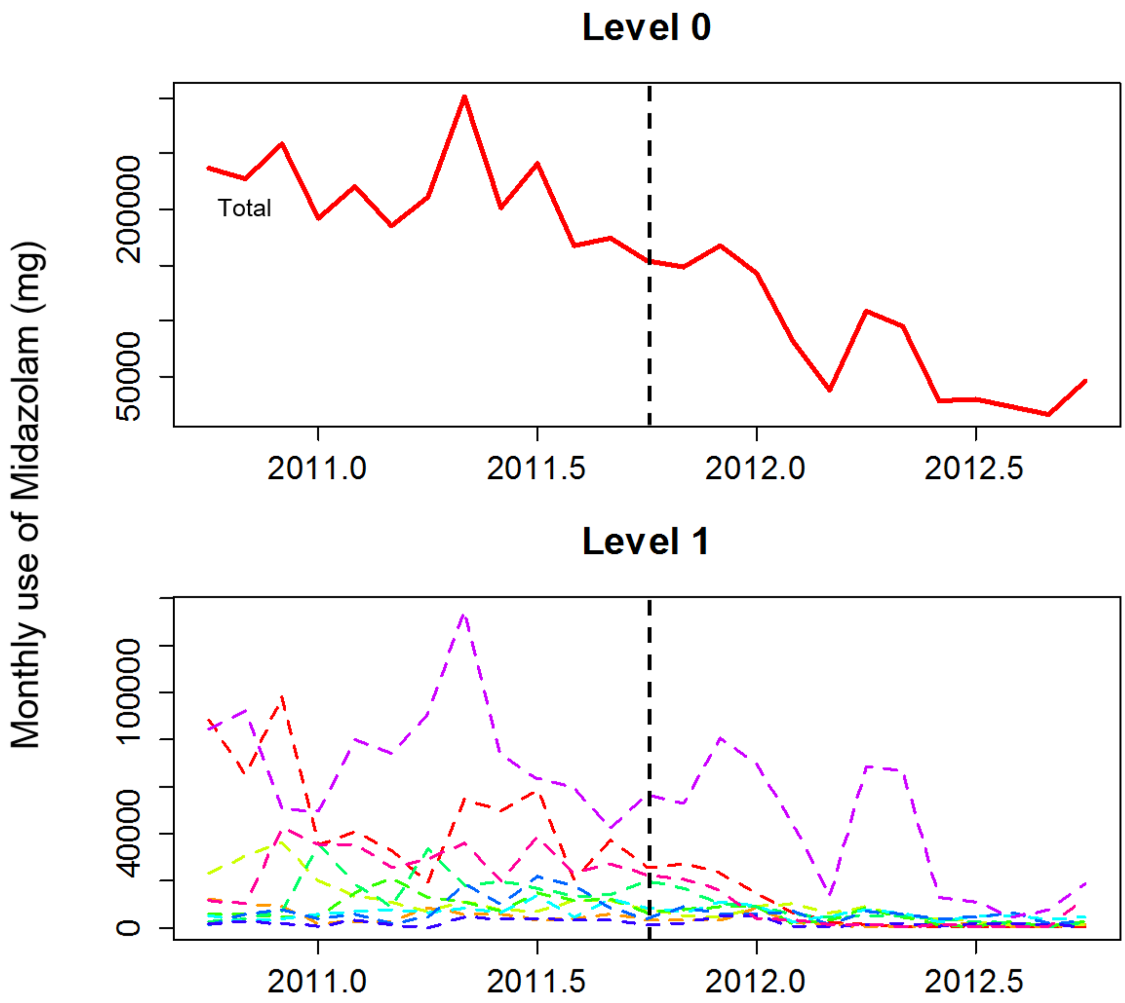

eFigure 6: **Results from the Hierarchical Time Series model for crude midazolam consumption.** Level 0 denotes the modeled time series using bottom-up method for the entire network. Level 1 denotes the independent time series for each one of the 10 units analysed. *Dashed black lines* represent when the intervention began.

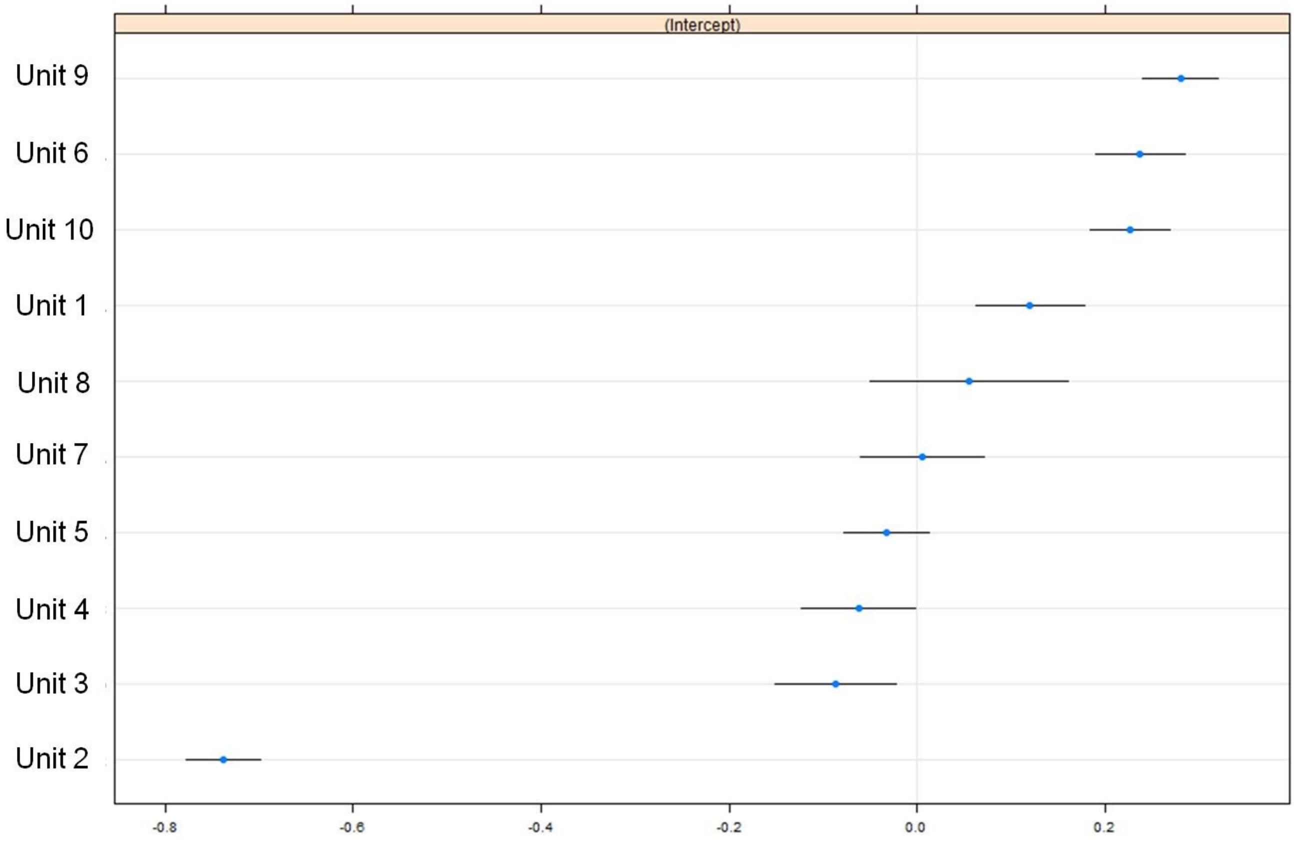

eFigure 7: **Plotting of the random intercepts per each unit from the generalized linear mixed model for the days under mechanical ventilation**.

References:

1. Amaral AC, Kure L, Jeffs A: **Effects of increasing compliance with minimal sedation on duration of mechanical ventilation: a quality improvement intervention**. *Crit Care* 2012, **16**(3):R78.
2. Wagner AK, Soumerai SB, Zhang F, Ross-Degnan D: **Segmented regression analysis of interrupted time series studies in medication use research**. *J Clin Pharm Ther* 2002, **27**(4):299-309.
3. Shardell M, Harris AD, El-Kamary SS, Furuno JP, Miller RR, Perencevich EN: **Statistical analysis and application of quasi experiments to antimicrobial resistance intervention studies**. *Clin Infect Dis* 2007, **45**(7):901-907.
4. Madden JM, Soumerai SB, Lieu TA, Mandl KD, Zhang F, Ross-Degnan D, organization Hm: **Effects of a law against early postpartum discharge on newborn follow-up, adverse events, and HMO expenditures**. *N Engl J Med* 2002, **347**(25):2031-2038.
5. Hyndman RJ, Ahmed RA, Athanasopoulos G, Shang HL: **Optimal combination forecasts for hierarchical time series**. *Computational Statistics & Data Analysis* 2011, **55**(9):2579–2589.
6. Chan J, Choy S: **Analysis of Covariance Structures in Time Series**. *Journal of Data Science* 2008, **6**(4):573-589.
7. Szyszkowicz M: **Use of generalized linear mixed models to examine the association between air pollution and health outcomes**. *Int J Occup Med Environ Health* 2006, **19**(4):224-227.
8. Bates D: **lme4: Mixed-effects modeling with R**: Springer; 2010.
9. Zhang D, Lin X: **Variance Component Testing in Generalized Linear Mixed Models for Longitudinal/Clustered Data and other Related Topics**. In: *Random Effect and Latent Variable Model Selection.* Edited by Dunson DB: Springer New York; 2008.
10. Goldstein H, Browne W, Rasbash J: **Partitioning Variation in Multilevel Models**. *Understanding Statistics* 2002, **1**(4):223-231.
